# Our Book Table

**Published:** 1884-06

**Authors:** 


### DEFERRED.

We have marked for our pages a number of interesting extracts from that valuable journal, "Archives of Pediatrics," but they have so far been crowded out by the press of other matter. If any one wishes to see them before we can reproduce them, as well as to get much other information of value to those practicing dentistry, let him subscribe for the journal. It is now published in New York.

### OUR BOOK TABLE.

ELEMENTARY PRINCIPLES OF ELECTRO THERAPEUTICS ; *for the use of Physicians and Students. One hundred and thirty-five illustrations:* by C. M. HAYNES, M. D. Chicago, Ill.: The McIntosh Galvanic and Faradic Battery Co. Octavo, pp. 426.

The use of electricity has become a part of the established practice of medicine, and the practitioner who has not some knowledge of its application is but partially fitted for his duties. In the light of the latest investigations, which seem to demonstrate that electricity is but one of the manifestations of the unit force, and that, save in its mode of expression it is identical with light, heat, nervous energy, etc., its importance as a curative agent may be comprehended. If, under the proper conditions each of these modes of force may be transformed into the other, electricity may be as essential to normal function as light, or heat, and an understanding of its proper application is a part of the necessary schooling of the successful physician.

In surgery, too, electricity is playing no unimportant part. The removal of nævi and many forms of neoplasm is often most readily performed by electrolysis, while in the treatment of strictures and other diseases of the urinary organs a good electrical apparatus has become essential. In dental medicine and surgery there is frequent necessity for its use, so that a text-book for reference and instruction should form a part of the library of every practitioner.

The book under notice amply fulfills every requirement. Although published by a manufacturing company, it is not disfigured by advertisements of their wares, but is devoted to the legitimate purpose of giving scientific information. The first part contains a succinct account of electrical laws so far as they are known. The different currents are explained, and their use in medicine illustrated, with explicit directions for their application. The principles that govern electrolysis are made plain, and the whole system of the therapeutics of electrical science is placed within the comprehension of all. There is a complete vocabulary of the scientific terms used, and altogether the book is one that can be heartily recommended to the profession.

NOTES ON DENTAL PRACTICE. By HENRY C. QUINBY, *Licentiate in Dental Surgery of the Royal College of Surgeons in Ireland, and member of the Odontological Societies of New York and London.* Philadelphia: The S. S. White Dental Manufacturing Co. 1884. Octavo, pp. 202.

This is another of the many works on dental practice that have been offered to the profession within a few years. It is plain and practical in matter, clear and concise in language, while in scope it treats of the mechanical aspect of most of the common diseases that the dentist is likely to meet in practice. The book is extremely provocative of thought and reflection, for the author is a man of positive views, and his fearlessness in the expression of them indicates one who is thoroughly convinced of the correctness of his position. But there is scarce a dentist of like settled and confident opinions who will not find on almost every page something to antagonize, and a reading of the book is like holding an argument with an intelligent and acute adversary. The benefits to be derived from its perusal greatly transcend those gained by the reading of an author who complacently pursues the regular beaten track of the common-place—that is, if he has his own ideas of dental practice—if he has not, the work is not intended for his eyes.

We have said that the book speaks of the mechanical treatment of dental diseases. There is very little of pathology in it, and yet less of therapeutics. Indeed, the author states in the preface that he has not attempted any physiological research, and he has perhaps placed himself beyond the reach of criticism in this respect by his naive confession. And yet we cannot comprehend how dental *practice* can be considered without taking into account the therapeutical agents that are necessary, absolutely essential, to its conduct. A successful practice cannot be carried on with forceps, excavators, pluggers, and other mechanical tools alone. In this respect, then, the book seems to an American practitioner at least lamentably deficient. We wish, too, that he had amended his nomenclature, and not called a tooth-pulp the "nerve."

We might go on interminably finding fault, for opportunities would not be lacking, but despite this must acknowledge that we like the sturdy positiveness of the man, and a perusal of his book has benefited us more than would the reading of an armful of the milk-and-water truisms of authors who fear to speak their mind lest they be antagonized by some one. But its profitable reading presupposes a fair knowledge of the subjects of which the author speaks.

The book is handsomely printed and bound, as indeed are all the works published by the S. S. White Dental-Manufacturing Co.

**The remainder of "Our Book Table" is necessarily deferred.**

